# Oral Zinc Supplementation Decreases the Serum Iron Concentration in Healthy Schoolchildren: A Pilot Study

**DOI:** 10.3390/nu6093460

**Published:** 2014-09-04

**Authors:** Naira Josele Neves de Brito, Érika Dantas de Medeiros Rocha, Alfredo de Araújo Silva, João Batista Sousa Costa, Mardone Cavalcante França, Maria das Graças Almeida, José Brandão-Neto

**Affiliations:** 1 Center for Health Sciences, Federal University of Rio Grande do Norte (UFRN), Av. Gal. Gustavo Cordeiro de Farias, s/n, CEP 59012-570, Natal, RN, Brazil; E-Mails: nairanbrito@yahoo.com.br (N.J.N.B.); eg76@hotmail.com (É.D.M.R.); alfredoas100@gmail.com (A.A.S.); 2 Department of Chemical Engineering, Center for Industrial Technology, UFRN, Av. Senador Salgado Filho, 3000, BR 101 km 92, Campus Universitário, CEP 59072-970, Natal, RN, Brazil; E-Mail: jbsc01@yahoo.com.br; 3 Department of Statistics, UFRN, Av. Senador Salgado Filho, 3000, BR 101 km 92, Campus Universitário, CEP 59072-970, Natal, RN, Brazil; E-Mail: mardonefranca@globo.com; 4 Department of Clinical and Toxicological Analyses, UFRN, Av. Gal. Gustavo Cordeiro de Farias, s/n, CEP 59012-570, Natal, RN, Brazil; E-Mail: mgalmeida84@gmail.com; 5 Department of Internal Medicine, UFRN, Av. Gal. Gustavo Cordeiro de Farias, s/n, CEP 59012-570, Natal, RN, Brazil

**Keywords:** oral zinc supplementation, serum zinc and iron, zinc and iron antagonism, iron status, schoolchildren

## Abstract

The recognized antagonistic actions between zinc and iron prompted us to study this subject in children. A convenience sample was used. Thirty healthy children between 8 and 9 years of age were studied with the aim of establishing the effect of a 3-mo oral zinc supplementation on iron status. Fifteen individuals were given a placebo (control group), and 15 were given 10 mg Zn/day (experimental group). Blood samples were collected at 0, 60, 120, 180 and 210 min after a 12-h overnight fast, before and after placebo or zinc supplementation. This supplementation was associated with significant improvements in energy, protein, fat, carbohydrate, fiber, calcium, iron, and zinc intake in accordance with the recommendations for age and sex. The basal serum zinc concentration significantly increased after oral zinc supplementation (*p* < 0.001). However, basal serum iron concentrations and area under the iron curves significantly decreased in the experimental group (*p* < 0.0001) and remained at the same level throughout the 210-min study. The values obtained for hemoglobin, mean corpuscular volume, ferritin, transferrin, transferrin saturation, ceruloplasmin and total protein were within normal reference ranges. In conclusion, the decrease in serum iron was likely due to the effects of chronic zinc administration, and the decrease in serum iron was not sufficient to cause anemia.

## 1. Introduction

Zinc is essential for many biological functions in all living organisms. Zinc deficiency, considered a nutritional disorder, has been associated with a variety of symptoms and diseases. This deficiency may be due to inadequate intake, malabsorption, excessive loss, increased demand, or other pathologies compromising its bioavailability [1].

Like zinc, iron is essential for humans, mainly for oxygen transport, hematopoiesis and central nervous system development. Serum iron is not an adequate parameter to diagnose iron deficiency because it exhibits analytical and biological limitations in assessing iron status. Inadequate dietary iron, physiological states and various pathologies can influence iron status testing [2].

The literature reveals that zinc and iron have been studied in children with respect to a number of clinical outcomes such as growth, infection, diarrhea, anemia, cognition and behavior [3,4]. Combined supplementation with zinc and iron has been recommended because zinc deficiency often coexists with iron deficiency in children from developing countries [5]. Zinc and iron have interactive effects that are most likely based on the chemical similarity between the two micronutrients. Thus, the inhibitory effect of zinc on iron absorption (and *vice* *versa*) may be due to the antagonistic action of zinc during the process of iron absorption from the gastrointestinal tract [6,7]. In this regard, divalent metal transporter 1 (DMT1) was previously described as the probable zinc and iron interaction site. DMT1 is located on the apical side of the small intestinal epithelium [8]. However, another study on this shared pathway focused on Zip14 as the potential locus of iron–zinc interactions [9].

Many tests are available for the determination of iron status. However, due to specimen integrity issues, analytical problems, biological and diurnal variations, and interpretation limitations, no gold standard exists [2]. Certain hematologic and biochemical parameters have been proposed to characterize iron status such as hemoglobin, serum ferritin, transferrin saturation, Zn-protoporphyrin, soluble transferrin receptors, and different indices or ratios between some of these parameters. Nevertheless, the diagnosis of iron deficiency or iron-deficiency anemia is confirmed only when two or more very low concentrations or abnormal hematologic and biochemical measurements of iron status are obtained [10]. Antunes *et al.* (2010) recently demonstrated that zinc supplementation reduced iron absorption but did not promote anemia in healthy children [11].

Accordingly, a need exists for more studies to establish the antagonistic effects of zinc on iron and the onset of anemia to evaluate the benefits in intervention programs. Additionally, zinc is administered in high doses and for long periods in many disorders, particularly in healthy children with growth deficiency, and in these conditions, attention to the onset of anemia is necessary. The aim of this study was to investigate whether a dose of 10 mg Zn/day for 3-mo might affect iron status in healthy children.

## 2. Materials and Methods

### 2.1. Subjects

A total of 30 healthy children of both sexes, aged 8–9 years, from four municipal schools in the northeastern city of Natal, Brazil, were studied. We studied healthy children because there are only rare studies in the literature regarding the effects of zinc on iron metabolism in this population. Many healthy children use oral zinc for growth and development. The published articles have only focused on debilitated children and those with zinc deficiency. Here, the inclusion criteria were healthy schoolchildren, as assessed clinically, anthropometrically and by laboratory assessment. The exclusion criteria included early pubarche, thelarche or menarche; acute, chronic, infectious or inflammatory diseases; and children who had undergone surgery or were using vitamin and mineral supplements.

### 2.2. Ethics Approval

The protocol was approved by the University Hospital Research Ethics Committee of the Federal University of Rio Grande do Norte (UFRN) (no. 542/11). All schoolchildren agreed to participate in the study and informed consent was obtained from their parents and guardians.

### 2.3. Experimental Design

**Figure 1:**
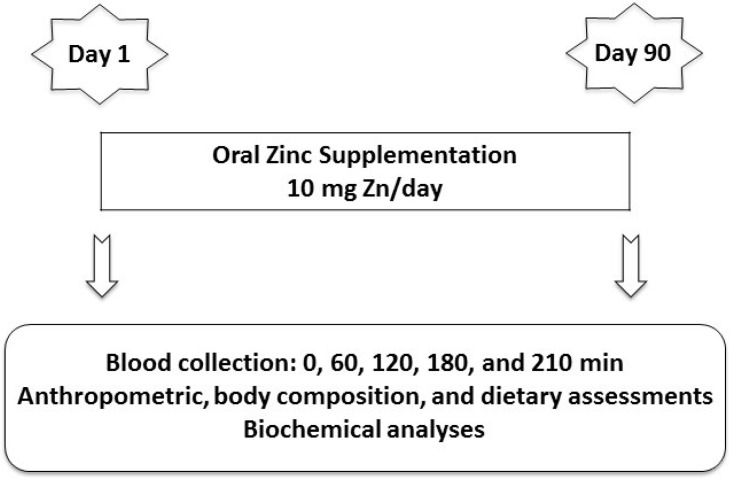
Experimental design of oral zinc administration in the control and experimental groups.

The volunteers were studied between 2011 and 2012. The study was characterized as a randomized, controlled, triple-blind trial with groups matched by age, formed by a process of non-probability sampling (convenience sample) in which the volunteers were divided into control and experimental groups. The study evaluated 15 schoolchildren as the control group to observe the serum zinc and iron profiles before and after the 3-mo oral placebo. The 3-mo period was established because it is already considered sufficient the study of zinc metabolism. The 15 schoolchildren in the experimental group were administered oral zinc, and serum zinc and iron were observed before and after the 3-mo zinc supplementation. The study began at 7:00 am and ended at 12:00 pm after a 12-h overnight fast (Figure 1). The subjects remained in the supine position throughout the test. An antecubital vein was punctured, and venous patency was maintained with a sterile, metal-free saline solution. Venipuncture was performed using plastic metal-free syringes without a tourniquet. Five basal blood samples were collected at 0, 60, 120, 180, and 210 min to better assess the children’s basal status (Figure 1). All tests began at 8:00 am. The zinc solution was not administered orally on the blood collection days. The subjects were monitored throughout the test, and professionals performed the sample collections. Zinc vials and zinc intake were monitored every 2 weeks by the same nutritionists during home visits. These same nutritionists performed anthropometric and nutritional assessments, and the same medical doctor (an endocrinologist) performed the clinical examination.

### 2.4. Anthropometric and Body Composition Assessments

All the schoolchildren were measured for body weight (kg) and height (cm) using an electronic balance (Balmak, BK50F, São Paulo, Brazil) and a stadiometer (Stadiometer Professional Sanny, American Medical do Brasil, São Paulo, Brazil), respectively. For weight measurement, the child stood on the scale wearing light clothing and without shoes. To measure height, the child remained standing without shoes or head gear and with heels together, arms extended along the body and with the body as upright as possible. The heels, buttocks, shoulders and head were aligned against the wall or vertical surface of the measuring equipment. The height and weight measurements were obtained three consecutive times by the same observer and then averaged. The observer was specifically trained for this purpose. The analysis of nutritional status was also based on the BMI-for-age [12,13].

### 2.5. Dietetic Assessment

Information on food intake was obtained at the beginning of the study and after 3-mo using food records from 3 nonconsecutive days (2 weekdays and 1 weekend day). Mothers were instructed to record each of the following about their child’s food intake: the time of each meal, the foods consumed, and their respective amounts. Energy, macronutrient, fiber, calcium, iron and zinc intake were calculated using NutWin software version 1.5 (UNIFESP, São Paulo, Brazil) [14]. Foods not included in the program were tabulated based on food chemical composition tables [15] or based on the nutritional information contained on the labels. Additionally, energy, protein, fat, carbohydrate, fiber, calcium, iron, and zinc were analyzed according to the recommendations for age and sex [16,17,18,19].

### 2.6. Oral Zinc Supplementation

The experimental group received 10 drops of zinc solution (10 mg Zn/day) added to milk or juice every morning at breakfast. The control group received an oral placebo (sorbitol 10%) under the same conditions. This 10 mg/day dose was considered physiologic and without risk of toxicity in children 8–9 years of age, mainly because it did not exceed the estimated average requirement (EAR) and the UL values.

### 2.7. Chemicals, Materials and Laboratory Procedures

Zinc sulfate heptahydrate (ZnSO_4_·H_2_O) was purchased from Merck (Darmstadt, Germany). The oral zinc solution (152.97 μmol Zn/day in the form of ZnSO_4_·7H_2_O) was prepared at the Pharmacotechnical Laboratory of the Department of Pharmacy, UFRN. Each drop contained 1 mg of elemental zinc. The placebo consisted of the same solution used to prepare the zinc solution. All the material used for the zinc and iron collections, separation, and storage was composed of metal-free propylene plastic. The tubes were purchased from BD-Becton (Dickinson and Company, Franklin Lakes, NJ, USA), and the pipettes were purchased from Bio-Rad (Bio-Rad Laboratories, Inc., Hercules, CA, USA).

After sample collection, the laboratory procedures were performed at the Multidisciplinary Laboratory of Chronic Degenerative Diseases. All micronutrient sample-handling procedures were performed according to international standards to avoid confounding factors [20]. The blood samples were placed in trace metal-free tubes without anticoagulants and stored for 120 min in a stainless steel incubator (FANEM 502, São Paulo, Brazil) until clot formation. Next, 500 μL of serum was collected with plastic trace metal-free pipettes and transferred to plastic tubes containing ultra-pure water (Milli-Q Plus, Millipore, Billerica, MA, USA) in the following concentrations: 2000 µL to dilute the serum for zinc (1:4) and 1500 μL for iron (1:3) analyses. The samples were held at −80 °C for subsequent analysis (Ultralow Freezer, Nuaire, MN, USA).

Serum zinc and iron samples were analyzed in triplicate within the same assay by atomic absorption spectrophotometry (SpectrAA-240FS, Varian, Australia) according to the manufacturer’s instructions. The zinc sensitivity was 0.01 μg/mL, the intra-assay coefficient of variation was 2.09%, and the normal reference range was 0.7–1.2 μg/mL. The iron sensitivity was 0.01 μg/mL, the intra-assay coefficient of variation was 2.79%, and the normal reference range was between 0.5 and 1.5 μg/mL.

The standard zinc solution (1000 mg/mL) was obtained by diluting Titrisol Zinc Standard (Merck, Darmstadt, Germany) in ultra-pure water. The standard iron solution (Titrisol Iron Standard, Merck, Darmstadt, Germany) was obtained in the same manner. The wavelength was 213.9 nm and 248.3 nm for zinc and iron, respectively. The lamp current was 10 mA, and all other procedures, such as calibrations and measurements, were performed in accordance with the manufacturer’s instructions.

The hematologic analyses, hematocrit, hemoglobin, and MCV were measured using standard clinical laboratory methods (Horiba ABX Diagnostics, Micros 60, Montpellier, France).

Biochemical parameters, such as transferrin, transferrin saturation and total protein, were measured by the colorimetric method in a biochemical analyzer (Dade Behring Dimension AR, Deerfield, IL, USA). Ferritin was measured by chemiluminescence (Immulite 2000, Diagnostic Products Corporation, Los Angeles, CA, USA), and ceruloplasmin was measured by nephelometry (Dade Behring BN II, Siemens Healthcare Diagnostics, Deerfield, IL, USA).

### 2.8. Statistical Analyses

Statistical analyses included the Shapiro-Wilk test to analyze the normality of all study data. A paired Student’s *t* test was used to compare the data obtained in iron profiles. Paired and unpaired Student’s *t* tests were used to analyze anthropometric index, energy and nutrient intake, basal serum zinc, area under the iron curves, and biochemical parameters. The Wilcoxon test was used for paired nonparametric testing or the Mann-Whitney test for unpaired nonparametric testing. Tukey’s multiple comparisons test was used to compare iron concentrations. Subsequently, to verify whether the results were true for the population studied, we used the sample size calculation for comparing two means (paired samples) as follows: *n* = (*Z* α + *Z* β)^2^ σ^2^_D_/δ^2^. Concentrations were expressed as the mean ± SEM. All comparisons were considered to be statistically significant at the 5% significance level. Analyses were performed using GraphPad Prism version 6.0 (GraphPad Software, Inc., San Diego, CA, USA).

## 3. Results

### 3.1. Subjects

Thirty healthy schoolchildren between 8 and 9 years of age were randomly assigned to the control and the experimental groups, and all completed the supplementation trial. The sample size of 30 schoolchildren was adequate for the conclusions obtained in this study given that for any value of α = 0.05, σ^2^ = 0.105034 and δ = −0.09, the sample size required would be *n* ≤ 15.

### 3.2. Anthropometric and Body Composition Assessments

According to BMI-for-age classification, all schoolchildren were eutrophic during the 3-mo study. The BMI was significantly different at the end of the study for both groups (Table 1).

**Table 1:** Body Mass Index (BMI) values and results for hemoglobin, mean corpuscular volume, ferritin, transferrin, transferrin saturation, ceruloplasmin and total protein in the control and experimental groups.

Parameter	Control			Experimental		
	Before	After	*p* Values	Before	After	*p* Values
BMI (kg/m^2^)	16.29 ± 0.40	16.60 ± 0.43	0.0424 *	16.06 ± 0.45	16.30 ± 0.45	0.0243 *
Hemoglobin (g/dL)	11.7 ± 0.18	11.7 ± 0.20	0.6829	11.6 ± 0.16	11.6 ± 0.17	0.7843
MCV (fL)	86.96 ± 0.99	84.88 ± 1.45	0.4973	85.55 ± 1.32	84.99 ± 1.06	0.7486
Ferritin (ng/mL)	29.9 ± 2.75	34.2 ± 3.53	0.3517	37.1 ± 3.25	34.0 ± 3.54	0.3415
Transferrin (mg/dL)	233 ± 7.22	238 ± 7.51	0.8904	229.1 ± 8.74	223.4 ± 6.22	0.2524
Transferrin saturation (%)	33.6 ± 1.12	32.6 ± 1.28	0.6224	34.7 ± 1.28	34.2 ± 1.36	0.7244
Ceruloplasmin (mg/dL)	31.2 ± 1.82	32.4 ± 1.53	0.5591	30.1 ± 1.58	27.6 ± 1.17	0.1367
Total protein (g/dL)	6.67 ± 0.26	6.49 ± 0.20	0.5511	6.54 ± 0.18	6.59 ± 0.18	0.8573

Note: Values are expressed as the mean ± SEM. Paired Student’s tests were used and Wilcoxon test for paired nonparametric test. * *p* values were significant for BMI in the control and experimental groups comparing before and after oral zinc supplementation.

### 3.3. Dietetic Assessment

Energy, protein, fat, carbohydrate, fiber, calcium, iron and zinc were normal according to the recommendations for age and sex, and showed a significant increase after oral zinc supplementation, indicating increased food consumption after the 3-mo study. Although fiber and calcium concentrations increased after oral zinc supplementation, they remained low in comparison to the recommendations by age and sex (Table 2).

**Table 2:** Energy and nutrient intake before and after oral zinc supplementation (experimental group) or placebo (control group) compared with recommended intakes for age and sex in 30 schoolchildren.

Parameter	Before Supplementation	After Supplementation	*p* Values	Reference Values
Energy (kcal)				6–9 years (boys): 1573–1978 kcal/day [16] 6–9 years (girls): 1428–1854 kcal/day [16]
Control group	1562 ± 48.02	1566 ± 47.70	0.3090	
Experimental group	1617 ± 55.52	1796 ± 58.30	0.0002 **	
Protein (g)				4–8 years (both sexes): 0.76 g/kg/day [18] 9–13 years (both sexes): 0.76 g/kg/day [18]
Control group	40.29 ± 0.55	40.56 ± 0.53	0.0292 *	
Experimental group	41.13 ± 0.68	47.06 ± 0.61	<0.0001 **	
Fat (g)				ND [18]
Control group	35.92 ± 0.62	36.11 ± 0.62	0.0145 *	
Experimental group	36.46 ± 0.49	45.09 ± 2.44	0.0015 **	
Carbohydrate (g)				100 g/day [18]
Control group	183 ± 2.61	183.4 ± 2.58	0.1232	
Experimental group	183.3 ± 4.56	180.8 ± 4.43	<0.0001 **	
Fiber (g)				4–8 years (both sexes): 25 g/day [18] 9–13 years (girls): 26 g/day [18] 9–13 years (boys): 31 g/day [18]
Control group	10.70 ± 0.28	11.09 ± 0.28	<0.0001 *	
Experimental group	10.56 ± 0.25	11.71 ± 0.25	<0.0001 **	
Calcium (mg)				4–8 years (both sexes): 800 mg/day [19] 9–13 years (both sexes): 1100 mg/day [19]
Control group	630.90 ± 28.29	628.40 ± 23.69	0.7377	
Experimental group	587.80 ± 17.13	648.10 ± 14.53	<0.0001 **	
Iron (mg)				4–8 years (both sexes): 4.1 mg/day [17] 9–13 years (boys): 5.9 mg/day [17] 9–13 years (girls): 5.7 mg/day [17]
Control group	8.69 ± 0.18	8.79 ± 0.13	0.1489	
Experimental group	8.76 ± 0.08	9.34 ± 0.09	<0.0001 **	
Zinc (mg)				4–8 years (both sexes): 4 mg/day [17] 9–13 years (both sexes): 7 mg/day [17]
Control group	6.24 ± 0.13	6.51 ± 0.12	<0.0001 *	
Experimental group	5.96 ± 0.11	6.50 ± 0.11	<0.0001 **	

Note: Values are expressed as the mean ± SEM. Reference 16 means = energy requirements during growth. Reference 17 means = estimated average requirement. Reference 18 means = estimated average requirement or not determinable (ND) or adequate intake. Reference 19 means = estimated average requirement. * *p* < 0.05 in the control group comparing before *versus* after placebo. ** *p* < 0.05 in the experimental group comparing before *versus* after zinc supplementation.

### 3.4. Zinc Parameters before and after Oral Zinc Supplementation

**Figure 2:**
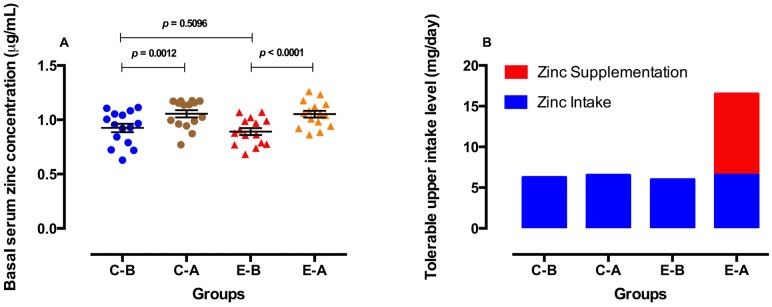
Basal serum zinc concentrations (**A**) and tolerable upper intake level of zinc (**B**) before and after 3 months of placebo (control group) or oral zinc supplementation (experimental group). C-B = Control-Before, C-A = Control-After, E-B = Experimental-Before, E-A = Experimental-After. Concentrations are expressed as the mean ± SEM. *p* values were calculated using paired and unpaired Student’s *t* tests. All comparisons were considered to be statistically significant at the 5% level (*p* < 0.05). **(**The previous figure was replaced by the current.).

Basal serum zinc concentrations increased in both the control and experimental groups, but the increase was greater in the experimental group (Figure 2A). However, the value of tolerable upper zinc intake level was higher in the experimental group after oral zinc supplementation, *i.e.*, 6.51 mg/day from zinc intake plus 10 mg/day from zinc supplementation. This was significantly different from the control group before and after placebo and the experimental group before zinc supplementation (Figure 2B). No side effects were observed at a dose of 10 mg Zn/day.

### 3.5. Iron Parameters before and after Oral Zinc Supplementation

**Figure 3:**
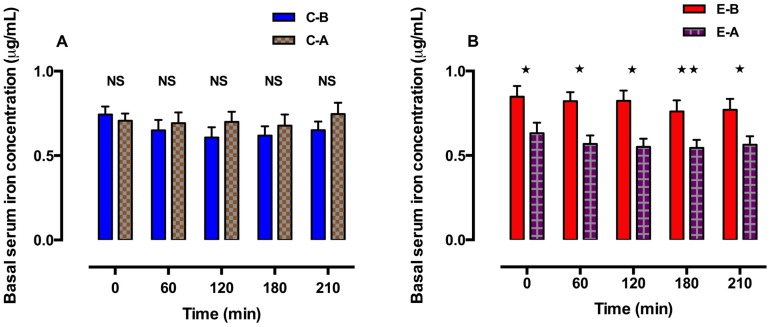
Basal serum iron concentrations in the control group (**A**) and experimental group (**B**) before and after 3 mo of placebo or oral zinc supplementation. Concentrations are expressed as the mean ± SEM. *p* values were calculated using Tukey’s multiple comparisons test. All comparisons were considered to be statistically significant at the 5% level (*p* < 0.05). NS = not significant. * *p* < 0.0001. ** *p* = 0.0007.

**Figure 4:**
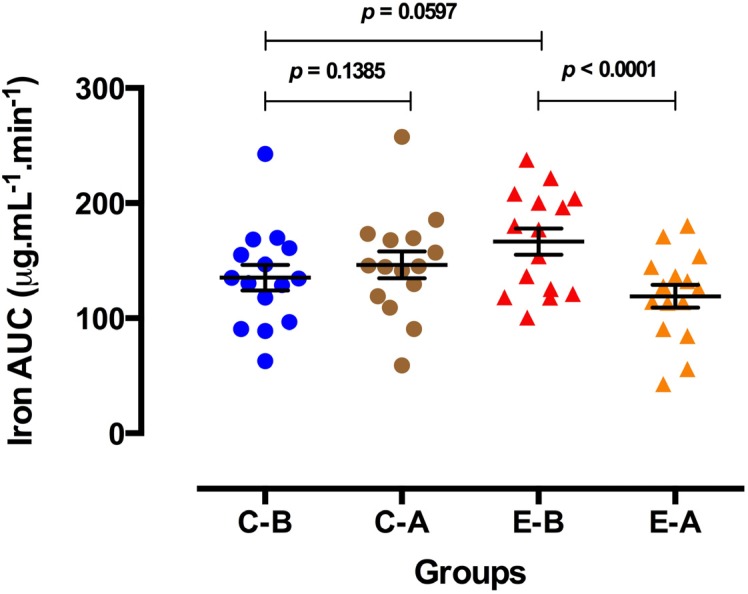
Area under the iron curves in the control and experimental groups before and after 3 mo of placebo or oral zinc administration. C-B = Control-Before, C-A = Control-After, E-B = Experimental-Before, E-A = Experimental-After. Concentrations are expressed as the mean ± SEM. *p* values were calculated using paired and unpaired Student’s *t* tests. All comparisons were considered to be statistically significant at the 5% level (*p* < 0.05).

Basal serum iron concentrations were not significantly different in the control group after receiving the placebo (Figure 3A). However, basal serum iron concentrations decreased significantly in the experimental group after oral zinc supplementation and remained so until the end of the 210 min of study (Figure 3B). All concentrations were in the normal reference range. Additionally, the area under the iron curves decreased after oral zinc supplementation, which was observed from 0 to 210 min in the experimental group (Figure 4).

### 3.6. Hematologic and Biochemical Analyses

The hematologic and biochemical parameters are shown in Table 1. All parameters were within the normal reference ranges.

## 4. Discussion

In a prior study conducted by our team, we suggested that an oral physiologic dose of 5 mg Zn/day decreased serum iron in healthy children without causing anemia [11]. This finding led us to expand the study by using a higher dose of elemental zinc (10 mg/day). The children in the present study exhibited the same clinical characteristics and were followed using the same methodological design as in the previous investigation.

Several hypotheses have been postulated to explain zinc–iron interactions based on zinc and iron uptake studies in many different cells, including enterocytes [9]. Zinc and iron have chemically similar absorption and transport mechanisms [1]. The quantitative consequences of these interactions will depend on the relative concentrations of nutrients ingested [21] and the graded concentrations of zinc and iron [22]. Antagonistic action occurs between zinc and iron when they are used in aqueous solutions, diets and in some cases of supplementation or fortification programs [21,23,24]. Conversely, iron supplementation may decrease plasma zinc concentrations in young women [25].

Interestingly, it has been shown that high doses of zinc in aqueous solutions impair iron absorption, while no effect was observed when zinc was added to meals [22,26,27]. This latter finding contrasts with our results because we demonstrated that oral zinc supplementation with meals could decrease serum iron concentrations in healthy schoolchildren. Moreover, 12 weeks of supplemental zinc (22 mg/day) in adolescent athletes reduced plasma iron and affected iron nutritional status [28]. Cofortification with zinc sulfate also decreases iron absorption in children [29].

In the presence of organic substances, zinc and iron are absorbed by different mechanisms, particularly when administered as supplements [21]. Emphasis has been given to DMT1 with respect to the absorptive pathway shared by both micronutrients [8]. However, the role of this transporter, as a shared pathway, remains controversial because it has a lower affinity for zinc [30,31]. In this context, Zip14, a transmembrane metal-ion transporter, is mechanically presented as the best interaction site between iron and zinc. Pinilla-Tenas *et al.* (2011) suggested that Zip14 stimulates the cellular uptake of zinc and nontransferrin-bound iron [32]. Iyengar *et al.* (2012) provided evidence that Zip14 appears to be the ideal transporter or site for iron and zinc, which provides a mechanistic view of the interactions between both ions during uptake in the enterocyte [9].

Although serum iron is considered an iron-status indicator, it is important to recognize other indicators such as hematologic and biochemical parameters. As such, we analyzed hematocrit, hemoglobin, MCV, ferritin, transferrin, transferrin saturation, ceruloplasmin and total protein. To date, no single publication has provided a comprehensive quantitative analysis of the parameters used to evaluate iron nutritional status. Although zinc supplementation decreased serum iron, it did not affect the aforementioned indicators of iron deficiency. Given that these indicators were within normal reference ranges, iron deficiency and iron-deficiency anemia were ruled out [23]. A likely explanation for this fact is that the decreased serum iron remained within the normal reference range. Additionally, all our schoolchildren were eutrophic during the 3-mo study, and food consumption was increased in the experimental group because energy, protein, fat, carbohydrate, fiber, calcium, iron and zinc showed a significant increase after oral zinc supplementation compared to the control group (Table 2). Moreover, the combined zinc intake (dietary + supplemental) did not exceed the tolerable upper intake level (UL) value for the age group between 9 and 13 years.

Furthermore, no impairment of the total plasma protein concentration was detected, which, when diminished, might alter serum zinc, iron, and transferrin. Corroborating our results, Antunes *et al.* (2010), in a sample of 11 children, found that serum zinc had a potential inhibitory effect on the serum iron profile, but the deficiency was not sufficient to cause anemia in the children studied [11]. These results agree with those of other studies. For instance, zinc supplementation did not adversely affect hemoglobin concentration in a meta-analysis [33]. Moreover, there were no differences in hemoglobin, serum ferritin, or serum transferrin receptors between children receiving zinc alone or placebo [5].

By contrast, a number of investigators reported a significant decline in hemoglobin in infants supplemented with zinc for 6 mo [3,34]. Additionally, young women supplemented with this micronutrient over a 70-day study period showed normal hemoglobin and a decrease in ferritin and transferrin saturation [35].

Additionally, zinc appears to interact with copper during absorption, which could affect the status of this micronutrient [28]. For example, in Wilson’s disease, zinc can induce copper binding metallothionein in duodenal enterocytes and in hepatocytes. Thus, copper absorption into the circulation is reduced, thereby decreasing the damaging effects of free copper [36]. This negative balance of copper in turn might affect iron transport and contribute to the risk of anemia in children [33]. In this context, we measured the concentration of ceruloplasmin, which was within the normal reference range. This parameter reinforces the finding that there was no apparent change in serum copper. Therefore, our results suggest that zinc supplementation does not appear to affect hemoglobin concentrations in healthy children. Overall, the relevance of this study is the demonstration that changes in iron metabolism are caused by physiologic zinc supplementation, even in healthy children*.*

The main limitation of our study was the difficulty in increasing the sample number of schoolchildren, hence the choice of the non-probability sampling method. Other limitations were (i) the extended time period in selecting the schoolchildren; (ii) standardization of the tests; (iii) the performance of 60 analyses; (iv) extensive dietary assessment and biochemical analysis; and (v) logistical complexity.

## 5. Conclusions

Our study suggests that chronic oral zinc supplementation in physiologic doses most likely inhibited iron absorption in healthy schoolchildren. The negative effect of zinc on serum iron did not promote anemia, although the duration of supplementation and the dose should be considered. These findings are preliminary and warrant additional study.
